# Association of Frailty Status and Dietary Patterns in a Nationally Representative Sample of United States Adults with Olfactory Dysfunction

**DOI:** 10.3390/nu14061238

**Published:** 2022-03-15

**Authors:** Varun Vohra, Evelyn M. Leland, Rodney J. Schlosser, Vidyulata Kamath, Nicholas R. Rowan

**Affiliations:** 1 Department of Otolaryngology-Head and Neck Surgery, Johns Hopkins University, Baltimore, MD 21287, USA; vvohra1@jh.edu (V.V.); eleland1@jhmi.edu (E.M.L.); 2 Department of Otolaryngology-Head and Neck Surgery, Medical University of South Carolina, Charleston, SC 29425, USA; schlossr@musc.edu; 3 Department of Psychiatry and Behavioral Sciences, Johns Hopkins University School of Medicine, Baltimore, MD 21287, USA; vkamath@jhmi.edu

**Keywords:** smell, nutrients, frailty, diet

## Abstract

Background: Olfactory dysfunction (OD) is a strong, independent predictor of frailty and mortality risk. This study evaluated the association of dietary patterns and frailty status in older adults with OD. Methods: This cross-sectional study utilized the 2013–2014 National Health and Nutrition Examination Survey. Dietary patterns (DPs) characteristic of OD were derived using exploratory factor analysis (EFA). Multiple logistic regressions adjusted for demographics and frailty risk factors assessed the association of DPs with two frailty metrics: the frailty index (FI) and physical frailty (PF). Results: EFA yielded six distinct DPs in persons with OD. The protein/selenium (OR 0.82 [95% CI 0.74–0.92], *p* = 0.041) and β-carotene/vitamin A DPs (OR 0.76 [95% CI 0.66–0.88], *p* = 0.028) were independently associated with frailty by FI. Only the protein/selenium DP (OR 0.82 [95% CI 0.74–0.92], *p* = 0.036) was associated with frailty by PF. No DPs were associated with either frailty measure in normosmic persons. Conclusions: Dietary patterns high in protein/selenium and β-carotene/vitamin A are associated with lower frailty prevalence in adults with OD. While the relationship between OD and frailty is likely multifaceted, these findings suggest that dietary patterns are uniquely associated with frailty in older adults with OD.

## 1. Introduction

Olfactory dysfunction (OD) is increasingly prevalent with age, with up to 62.5% of older adults aged 80 years or older demonstrating evidence of OD [1]. An increasing body of literature suggests that OD is a harbinger of both frailty and mortality [2,3,4,5,6,7,8]. Frailty is a unique syndrome, distinct from age, characterized by reduced physiologic reserve and increased vulnerability to adverse health outcomes. As a clinical tool, frailty reliably predicts increased falls, hospitalization, dependence, and mortality [9]. Higher rates of frailty have also been associated with increased healthcare costs through greater numbers of consultations, increased hospital admissions, and longer hospital stays [10]. Given the increasing relevance of frailty, which may be present in up to 12–24% of community-dwelling older adults globally, there is considerable impetus to improve the recognition of individuals at risk, identify modifiable risk factors to prevent progression of frailty, and aid in the treatment of frail older adults [11].

As frailty is broadly recognized as a state of increased vulnerability, multiple instruments for the assessment of frailty have been developed. Among these, physical frailty (PF) and the frailty index (FI) are two foundational metrics that remain fundamental in the understanding and measurement of frailty. PF is defined as a syndromic concept that includes unintentional weight loss, low energy, slow gait, reduced grip strength, and reduced physical activity [12]. Meanwhile, FI is a deficit accumulation model that operationalizes frailty by assessing the fraction of a set of deficits present in an individual. Though similar by name, these two approaches are not interchangeable [13]. PF and FI have been shown to predict frailty with similar prevalence, though the populations captured by the instruments are different, highlighting their distinct utilities in practice [14]. Though PF and the FI have helped to define an entire field of study, it is imperative that frailty research build upon these founding principles towards actionable investigations, such as clinical interventions and practice modifications.

The unique chemosensory function of olfaction may lie at the intersection of both identification and treatment of frail individuals. Though several associative studies have established that OD is strongly and independently predictive of frailty and mortality, there is little understanding of the mechanisms underlying these associations [2,3,4,5,6,7,8]. Changes in nutritional intake represent one potential pathway linking OD to adverse health outcomes. While the exact relationship between OD and nutritional status is complex, OD has been linked to decreased food enjoyment, abnormal nutrient sensing [15,16], and possibly altered energy homeostasis and metabolism [9]. Previous studies examining the specific dietary changes associated with OD have largely focused on single nutrients and foods, noting a relationship between OD and lower intakes of fat, protein, folate, magnesium, and phosphorous [17,18]. These findings were supported by a recent population-level study reporting a correlation between measured OD and decreased intake of multiple nutrients, while subjective OD was associated with a lower score on a metric of healthy dietary patterns [19]. Similar studies characterizing dietary associations with frailty have found links with protein, β-carotene, cholesterol, and vitamin D [20,21].

While single-nutrient studies have served as the foundation of dietary investigations of these outcomes, this methodology may overlook the complexity of dietary intake. Due to the high intercorrelation and synergistic effects of nutrient intakes, isolating individual effects is less meaningful. Moreover, prior clinical interventions based on single nutrients and foods have been less efficacious than dietary pattern interventions in reducing disease risk [22]. To address this challenge, data-driven approaches such as principal components analysis (PCA) and exploratory factor analysis (EFA) have been employed to characterize dietary patterns [23]. This approach recognizes that nutrients are consumed in combinations and examines the correlations between all nutrients in an individual’s diet. Subsequently, this large number of nutrients/dietary intakes is reduced into a smaller set of dietary patterns, or factors, that characterize highly correlated nutrient intakes. Critically, the goal of this method is to reduce the number of dietary variables while accounting for the greatest possible variance in a given population’s diet.

To investigate the relationship between olfactory dysfunction, diet, and frailty, we aimed to (1) characterize the dietary patterns of older adults with OD in a nationally representative sample and (2) investigate the association of these dietary patterns with frailty. Given that the association between frailty and OD has been previously described in this sample, we focused our analyses on diet and frailty within the OD group [2]. Importantly, we sought to operationalize frailty using both the physical frailty phenotype and deficit accumulation approaches. We hypothesized that examining both frailty measurements in a complementary fashion would offer a more comprehensive investigation into the dietary basis of frailty risk. Findings from this study may provide important insight into the mechanistic underpinnings between OD and frailty, bolstering the role of OD as an actionable biomarker in preventing adverse health outcomes.

## 2. Materials and Methods

### 2.1. Population

The study population for this analysis included subjects from the 2013–2014 National Health and Nutrition Examination Survey (NHANES) cycle aged 40 years and older who completed olfactory testing and the dietary interview. This analysis focused on persons with identified OD (as outlined below), but normosmic persons were also included in the analysis as a control population. 

### 2.2. Olfactory Assessment 

The 2013–2014 NHANES assessed olfaction with the 8-item Pocket Smell Test (PST, Sensonics, Inc., Haddon Heights, NJ, USA) as previously described [24]. Briefly, the test assessed olfactory identification with eight suprathreshold separate odors in a forced-choice format. Participants were scored on a scale from 0 to 8 and considered to have OD if 3 or more out of 8 odorants were identified incorrectly.

### 2.3. Dietary Recall

The 2013–2014 NHANES contained a dietary intake interview that assessed dietary intake on three nonconsecutive 24-hour periods. A registered dietician provided guidance on maintaining a detailed food record. The “Total Nutrient Intakes” files from NHANES 2013–2014 were used. Nutrient data and dietary data-specific sample weights for each dietary interview day were averaged for all survey-adjusted statistical analyses.

### 2.4. Frailty

Frailty was assessed through both the PF and deficit accumulation approaches (FI). For PF classification, a modified Fried frailty model was employed, as previously described for NHANES data [25]. Briefly, PF was defined based on the following five measurements: weakness, low physical activity, exhaustion, slow walking speed, and unintentional weight loss. Similarly, FI was operationalized using a 39-item frailty index (FI), as previously described [2]. Each of the 39 variables was coded as 0 (no deficit) or 1 (deficit present). All variables included in the FI are included in Table S1. FI was recorded as a score from 0 (total fitness) to 1 (total frailty). In each case, the FI score was calculated as a continuous variable by taking the sum of all variables over the total number of available values. In addition, the FI values were also divided dichotomously into frail and not frail categories, with non-frail ≤0.21 and frail ≥0.21.

### 2.5. Statistical Analysis

Statistical analyses were conducted using R statistical software (version 1.3.1093, r-project.org). All statistical analysis followed CDC guidelines for complex, multistage survey analysis in NHANES, including the appropriate weights for all analyses. Dietary patterns were derived using exploratory factor analysis with a principal component extraction method. Nutrient data were verified for factorability based on the Kaiser–Meyer–Olkin test (KMO = 0.88) for sample adequacy and by Bartlett’s test of sphericity (*p* < 0.001) to ensure correlation between nutrients. The number of factors was chosen based on parallel analysis and visual inspection of the scree plot, a graphical representation of eigenvalues for each dietary pattern [26]. The initial factor solution was rotated using orthogonal varimax rotation to improve interpretability and attain a simpler structure. Pattern adherence scores, or factor scores, were calculated as the linear combination of factor loadings and standardized nutrient intakes for each dietary pattern. Higher scores indicate greater adherence to a given dietary pattern. Dietary patterns were named and referred to in the text based on the two highest factor loadings.

For bivariate analyses, pattern adherence scores were categorized into tertiles. Categorical variables were compared using survey-weighted chi-square tests with the Scott–Rao correction. Continuous variables were compared using survey-weighted Kruskal–Wallis tests. For multivariable analyses, pattern adherence scores were analyzed continuously. Adjusted logistic regression models were used to assess the relationship between dietary patterns and the risk of frailty. Nutrient intakes were adjusted for energy using the residual method [27]. Models were further adjusted for age, gender, race, income-to-poverty ratio, body mass index (BMI), total energy intake, and smoking status.

## 3. Results

### 3.1. Participant Demographics

The average age of the study population was 69.6 ± 0.71 years (Table 1), and the average age of the normosmic control group was 63.8 ± 0.3. The participants were 58% male. In participants with OD, frailty was more prevalent based on the FI score (42%) than the PF score (10.8%). All frail individuals by PF were also classified as frail by FI.

Within the OD population, comparisons between frail and non-frail groups based on the FI scores indicated several significant differences (Table 1). Frail participants were more likely to be older (71.08 ± 0.89 vs. 68.0 ± 0.91, *p* < 0.001), female (48% vs. 37%, *p* < 0.001), and have a lower income-to-poverty ratio (2.36 ± 0.18 vs. 2.99 ± 0.22, *p* = 0.04). With regard to PF (Table 2), frail participants had a significantly higher BMI (33.9 ± 1.67 vs. 28.52 ± 0.52, *p =* 0.04). Frail participants had a significantly lower caloric intake than non-frail participants, as indicated by both the FI (1663.7 ± 79.3 vs. 1886.9 ± 47.18, *p* = 0.017) and PF (1559.83 ± 125.24 vs. 1821.2 ± 42.13, *p* = 0.039) classifications.

### 3.2. Dietary Patterns and Olfaction

**Figure 1:**
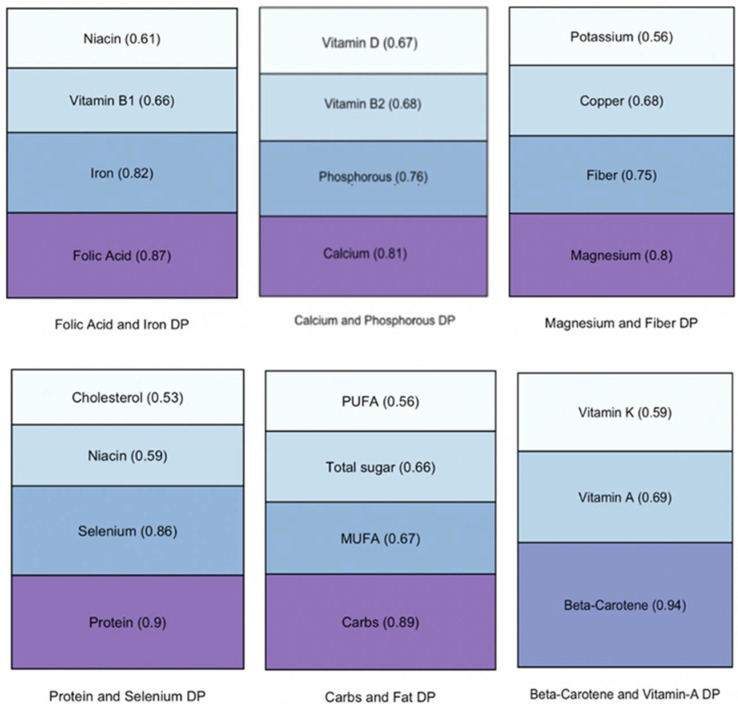
Composition of dietary patterns by largest component weights. Note MUFA = monosaturated fatty acid, PUFA = polysaurated fatty acid, DP = dietary pattern.

Factor analysis yielded 6 dietary patterns (DPs) from the original 28 nutrition variables (Table S2). Together, these six DPs accounted for 63.4% percent of the variance in the nutritional data. The highest loadings for each DP are represented in Figure 1.

Fully adjusted multivariable linear regressions comparing DP scores between dysosmic and normosmic groups (Table 3, Model 2) indicated that DP 3 (magnesium and fiber; *p* = 0.01), DP 4 (protein and selenium; *p* = 0.006), and DP 6 (β-carotene and vitamin A; *p* < 0.001) were significantly lower in the dysosmic group.

### 3.3. Dietary Patterns and Frailty: Bivariate Analysis

In dysosmic participants, bivariate analysis of DP scores and frailty revealed DP 2 (calcium and phosphorous; *p* = 0.018), DP 3 (magnesium and fiber; *p* = 0.004), DP 4 (protein and selenium; *p* < 0.01), and DP 6 (β-carotene and vitamin A; *p* < 0.001) were significantly associated with frailty by the FI score (Table 4). On the other hand, only DP4 (protein and selenium; *p* = 0.03) was significantly associated with frailty by PF.

### 3.4. Dietary Patterns and Frailty: Multivariate Analysis 

Multiple logistic regression models of the FI, adjusted for age, gender, race, BMI, income-to-poverty ratio, energy intake, and smoking status, yielded two significant DPs (Table 5). Specifically, DP4 (protein and selenium, OR 0.82 [95% CI 0.74–0.92], *p* = 0.041) and DP6 (β-carotene and vitamin A, OR 0.75 [95% CI 0.65–0.86], *p* = 0.027) were independently associated with FI. Similar models for PF indicated only DP4 (protein and selenium) as being significantly associated with frailty risk (OR 0.76 [95% CI 0.66–0.88], *p* = 0.037). An additional analysis exploring the role of gustatory dysfunction indicated no significant changes to these results (Table S3). Lastly, analogous bivariate and multivariate analyses of the relationship between DPs and frailty yielded no significant associations in a normosmic reference group (Table S4). 

Given the higher prevalence of identified frailty under the FI classification, the authors sought to better characterize the diets of participants who had contrasting classifications under the two frailty definitions. A subanalysis of participants that were categorized as frail using FI but non-frail per PF was conducted (Table S5). Adjusted multiple logistic regressions models (Table 6) revealed only DP6 (β-carotene and vitamin A, OR = 0.76 [95% CI 0.65–0.90], *p* = 0.043) as a significant dietary pattern for frailty risk in this subgroup.

## 4. Discussion

In this cross-sectional analysis of a nationally representative population, we derived dietary pattern (DP) characteristics of OD and sought to evaluate whether these unique DPs were independently associated with frailty. After adjusting for age, gender, race, income-to-poverty ratio, BMI, smoking, and total caloric intake in adults with dysosmia, DP4 (protein and selenium) and DP6 (β-carotene and vitamin A) were significantly associated with FI, while only DP4 was associated with the phenotypic model, PF. Specifically, DP4 and DP6 were independently and inversely associated with the risk of frailty such that lower adherence to these dietary patterns indicated a higher risk of frailty in older adults with OD. For both measures of frailty, there were no significant associations with DPs in a normosmic reference group, suggesting that this association between diet and frailty is unique to persons with OD.

The results of this study demonstrate that DP4 (protein and selenium) is strongly associated with both measures of frailty in dysosmic participants. DP4 was characterized by high consumption of protein, selenium, niacin, and cholesterol. Single dietary items fitting this DP generally include meats and seafood. The role of inadequate protein intake in frailty incidence has been well documented and has been associated with muscle wasting and atrophy in older adults [28,29]. Similarly, low selenium intake has been linked with poor grip strength and overall muscle strength [30,31]. On this basis, DP4 appears to be associated with sarcopenic elements of frailty, such as walking speed, weakness, and exhaustion, which are likely closely linked with changes in the dietary components of muscular health [32]. Additionally, DP4 showed negative weightings for total sugar and carbohydrates, suggesting that a combination of high protein/selenium intake coupled with low total sugar intake is protective for frailty risk. 

Overall, foods matching the nutritional characteristics of DP6 (β-carotene and vitamin A) consist largely of fruits and vegetables. In considering the role of DP6 in the risk of frailty, these results support previous findings that a higher intake of micronutrients, including β-carotene, vitamin A, and vitamin C, is associated with a lower prevalence of frailty [33,34,35]. The two largest loadings in DP6 were closely related as carotenoids, β-carotene (provitamin A), and its downstream product, vitamin A. In line with prior studies that have suggested a link between oxidative stress and frailty, higher β-carotene intake may be protective due to its antioxidant activity [36,37]. Kochlik et al. found that compared with frail subjects, healthy participants had significantly higher levels of several antioxidants, including γ-tocopherol, α-carotene, β-carotene, and lycopene, while frail individuals had significantly higher concentrations of oxidative stress biomarkers [38]. Notably, lower intakes of vitamin A, β-carotene, and fiber have also been linked with the risk of periodontal disease in older individuals, suggesting a vicious cycle contributing to the “anorexia of aging” that has been associated with frailty [39].

Moreover, in this population, FI was found to be a more sensitive measure of frailty than PF. Given these differences, we examined those individuals that were frail by the FI classification but not identified as frail by PF. Expectedly, this subgroup was younger and had a lower average FI than the full sample of frail individuals. This agrees with previous work demonstrating that, in contrast to extremely older adults (>72 years old), adults aged 65–72 years old were more likely to be classified as frail by FI but non-frail by PF, and associations with mortality and age were stronger when utilizing the PF metrics [14]. Interestingly, only DP6 was significantly associated with FI in this subgroup. This finding suggests that DP6 may be associated with either earlier stages of frailty or uniquely associated with deficit accumulation.

While there is strong evidence for the relationship between OD and frailty, mechanisms underlying this relationship are likely convoluted and remain to be elucidated. OD is increasingly prevalent in older adults, with up to a quarter of community-dwelling older adults meeting criteria for frailty, and represents a novel, accessible, and potentially modifiable biomarker of frailty [11,40]. Importantly, the current study is cross-sectional and thus cannot provide causal insight into the relationship between diet and olfaction. Nonetheless, one intuitive explanation is that dietary changes associated with OD are an active component of frailty incidence. The findings of this study may support this hypothesis as DP4 (protein and selenium) and DP6 (β-carotene and vitamin A) had no association with frailty in the normosmic reference group. However, reverse causality between OD and diet is also possible. For example, it is also plausible that vitamin A deficiency may be a risk factor for OD [41]. Alternatively, OD may be representative of central nervous system dysfunction and accelerated brain aging, and the DPs found in this study may instead function in a neuroprotective role [42,43,44]. Several other additional theories may also exist to explain this relationship unique to individuals with OD. 

Though further investigations are needed to understand the relationship between OD and frailty, these findings offer an opportunity to explore future interventions aimed at the mitigation of the harmful implications of frailty. For instance, the use of brief olfactory tests such as those used in the NHANES assessments could serve as screening measures to identify older persons at risk. Given the broad prevalence of olfactory dysfunction in the population, stemming from a variety of causes (e.g., Alzheimer’s disease, Parkinson’s disease, post-traumatic olfactory loss, chronic rhinosinusitis) dietary counseling reflective of protective components of identified DPs, such as protein, selenium, and antioxidants may be a low-risk intervention to mitigate frailty. Moreover, there may be additional opportunities to focus on interventions for the management of smell loss and associated complications (e.g., depression, safety risks).

Though this study provides strong evidence for a dietary association linking OD and frailty in a nationally representative sample, there are notable limitations. Primarily, due to the data-driven approach of the dietary data analysis in this study, it will be important to validate these associations in additional populations with future research directed at characterizing both dietary habits and olfactory function across time. Specifically, it would be valuable to examine longitudinal, dose-dependent changes in OD and pattern adherence scores to identify the temporality of dietary changes. Moreover, there are additional person-level differences, such as alternate etiologies of OD or odor-specific anosmias that could not be accounted for in this dataset. Despite these limitations, this study provides important insight into the association between OD and frailty and ultimately offers a framework for future prevention and intervention measures to address frailty risk.

## 5. Conclusions

Dietary patterns characteristic of older adults with OD, particularly diets high in protein/selenium and β-carotene/vitamin A, are associated with reduced risk of frailty in persons with OD. The relationship between OD and frailty is multifaceted, but dietary changes may be a potential pathway underlying this relationship. These findings may provide a framework for future interventions to mitigate the harmful implications of frailty.

## Figures and Tables

**Table 1:** Demographics of frail and non-frail older adults with olfactory dysfunction—frailty index.

	Total (*n* = 350)	Frail (*n* = 155)	Non-Frail (*n* = 195)	*p*-Value
Age	69.6 ± 0.71	71.8 ± 0.89	68.0 ± 0.0.91	<0.001 ***
Gender				<0.001 ***
Male	203 (0.58)	80 (0.52)	123 (0.63)	
Female	147 (0.42)	75 (0.48)	72 (0.37)	
Body mass index	29.7 ± 0.41	30.5 ± 0.89	29.05 ± 0.4	0.52
Race				
Non-Hispanic White	163 (0.47)	83 (0.54)	80 (0.41)	0.431
Mexican American	35 (0.1)	16 (0.1)	19 (0.1)	
Other Hispanic	35 (0.1)	11 (0.07)	24 (0.12)	
Non-Hispanic Black	86 (0.25)	36 (0.23)	50 (0.26)	
Non-Hispanic Asian	27 (0.08)	7 (0.05)	20 (0.1)	
Other Race	4 (0.01)	2 (0.01)	2 (0.01)	
Income-to-poverty ratio	2.71 ± 0.16	2.36 ± 0.18	2.99 ± 0.22	0.04 *
Current smoker				0.147
No	303 (0.87)	132 (0.85)	171 (0.88)	
Yes	47 (0.13)	23 (0.15)	24 (0.12)	
Energy (kcal)	1790.8 ± 46.7	1663.7 ± 79.3	1886.9 ± 47.18	0.017 *
Frailty index score	0.21 ± 0.12	0.34 ± 0.013	0.12 ± 0.05	<0.001 ***

*** (*p* < 0.001), * (*p* < 0.05).

**Table 2:** Demographics of frail and non-frail older adults with olfactory dysfunction—physical frailty.

	Frail (*n* = 38)	Non-Frail (*n* = 312)	*p*-Value
Age	68.76 ± 1.58	69.7 ± 0.84	0.7
Gender			0.067
Male	14 (0.37)	189 (0.61)	
Female	24 (0.63)	123 (0.39)	
Body mass index	33.9 ± 1.67	28.52 ± 0.52	0.04 *
Race			0.32
Non-Hispanic White	21 (0.55)	142 (0.46)	
Mexican American	3 (0.08)	32 (0.1)	
Other Hispanic	1 (0.03)	34 (0.11)	
Non-Hispanic black	11 (0.29)	75 (0.24)	
Non-Hispanic Asian	1 (0.03)	26 (0.08)	
Other Race	1 (0.03)	3 (0.01)	
Income-to-poverty ratio	2.35 ± 0.36	2.78 ± 0.2	0.3
Current smoker			0.057
No	29 (0.76)	274 (0.88)	
Yes	9 (0.24)	38 (0.12)	
Energy (kcal)	1559.83 ± 125.24	1821.2 ± 42.13	0.039 *
Frailty phenotype score	0.39 ± 0.01	0.19 ± 0.01	<0.001 ***

*** (*p* < 0.001), * (*p* < 0.05).

**Table 3:** Multivariable linear regression analyses examining the association between dietary patterns and olfactory function.

	Model 1 ^a^: β-Estimate [95% CI]	Model 2: β-Estimate [95% CI]
DP 1 (folate and iron)	−0.19 [−0.32 to −0.07] **	−0.11 [−0.24 to 0.01]
DP 2 (calcium and phosphorus)	−0.16 [−0.29 to −0.04] *	−0.103 [−0.23 to 0.02]
DP 3 (magnesium and fiber)	−0.25 [−0.37 to −0.12] ***	−0.16 [−0.29 to −0.04] *
DP 4 (protein and selenium)	−0.23 [−0.35 to −0.1] ***	−0.18 [−0.3 to −0.05] **
DP 5 (carbs and fat)	−0.14 [−0.27 to −0.01] *	−0.096 [−0.22 to 0.03]
DP 6 (β-carotene and vitamin A)	−0.19 [−0.32 to −0.06] **	−0.22 [−0.34 to −0.09] ***

AOR: adjusted odds ratio; CI: confidence interval; DP: dietary pattern. ^a^ Normosmic group served as the reference. Model 1 was adjusted for age, gender, race, and, income-to-poverty ratio. Model 2 included body mass index, energy, and smoking, in addition to all covariates from Model 1. *** (*p* < 0.001), ** (*p* < 0.01), * (*p* < 0.05).

**Table 4:** Association between dietary patterns and frailty in adults with olfactory dysfunction.

	Frailty Index			Physical Frailty		
	Frail (*n* = 155)	Non-Frail (*n* = 195)	*p*-Value	Frail (*n* = 38)	Non-Frail (*n* = 312)	*p*-Value
DP 1 (folate and iron)			0.06			0.36
Low	69 (0.45)	70 (0.36)		19 (0.5)	120 (0.38)	
Middle	48 (0.31)	65 (0.33)		11 (0.29)	102 (0.33)	
High	38 (0.25)	60 (0.31)		8 (0.21)	90 (0.29)	
DP 2 (calcium and phosphorous)			0.018 *			0.38
Low	74 (0.48)	73 (0.37)		21 (0.55)	126 (0.4)	
Middle	41 (0.26)	65 (0.33)		8 (0.21)	98 (0.31)	
High	40 (0.26)	57 (0.29)		9 (0.24)	88 (0.28)	
DP 3 (magnesium and fiber)			0.004 **			0.06
Low	71 (0.46)	66 (0.34)		19 (0.5)	118 (0.38)	
Middle	52 (0.34)	61 (0.31)		15 (0.39)	98 (0.31)	
High	32 (0.21)	68 (0.35)		4 (0.11)	96 (0.31)	
DP 4 (protein and selenium)			<0.01 **			0.03 *
Low	88 (0.57)	77 (0.39)		25 (0.66)	140 (0.45)	
Middle	35 (0.23)	65 (0.33)		8 (0.21)	92 (0.29)	
High	32 (0.21)	53 (0.27)		5 (0.13)	80 (0.26)	
DP 5 (carbs and fat)			0.07313			0.12
Low	66 (0.43)	74 (0.38)		20 (0.53)	120 (0.38)	
Middle	50 (0.32)	70 (0.36)		13 (0.34)	107 (0.34)	
High	39 (0.25)	51 (0.26)		5 (0.13)	85 (0.27)	
DP 6 (β-carotene and vitamin A)			<0.001 ***			0.06
Low	77 (0.5)	70 (0.36)		21 (0.55)	126 (0.4)	
Middle	44 (0.28)	53 (0.27)		13 (0.34)	84 (0.27)	
High	34 (0.22)	72 (0.37)		4 (0.11)	102 (0.33)	

DP: dietary pattern. *** (*p* < 0.001), ** (*p* < 0.01), * (*p* < 0.05).

**Table 5:** Multiple logistic regression analyses ^a^ examining the association between dietary patterns and frailty in older adults with olfactory dysfunction.

	Frailty Index			Physical Frailty		
	AOR	95% CI	*p*-Value	AOR	95% CI	*p*-Value
DP 1 (folate and iron)	0.93	0.88 to 0.98	0.07	0.97	0.81 to 1.17	0.79
DP 2 (calcium and phosphorus)	0.9	0.85 to 0.96	0.045	0.95	0.86 to 1.05	0.41
DP 3 (magnesium and fiber)	0.89	0.82 to 0.96	0.06	0.91	0.78 to 1.06	0.31
DP 4 (protein and selenium)	0.82	0.74 to 0.92	0.041 *	0.76	0.66 to 0.88	0.037 *
DP 5 (carbs and fat)	0.88	0.73 to 1.06	0.27	0.84	0.66 to 1.07	0.26
DP 6 (β-carotene and vitamin A)	0.75	0.65 to 0.86	0.027 *	0.84	0.62 to 1.15	0.36

AOR: adjusted odds ratio, CI: confidence interval, DP: dietary pattern. ^a^ Models were adjusted for age, gender, race, BMI, income-to-poverty ratio, energy, and smoking. * (*p* < 0.05).

**Table 6:** Subgroup classified as frail by FI and robust by FP—multiple logistic regression analyses ^a^ examining the association of dietary patterns with frailty in adults with OD.

	AOR	95% CI	*p*-Value
DP 1 (folate and iron)	0.92	0.83 to 1.03	0.23
DP 2 (calcium and phosphorus)	0.91	0.81 to 1.01	0.19
DP 3 (magnesium and fiber)	0.89	0.81 to 0.98	0.1
DP 4 (protein and selenium)	0.92	0.82 to 1.01	0.18
DP 5 (carbs and fat)	0.95	0.83 to 1.08	0.46
DP 6 (β-carotene and vitamin A)	0.76	0.65 to 0.90	0.043 *

AOR: adjusted odds ratio, CI: confidence interval, DP: dietary pattern. ^a^ Models were adjusted for age, gender, race, BMI, income-to-poverty ratio, energy, and smoking. * (*p* < 0.05).

## Data Availability

The publicly available National Health and Nutrition Examination Survey (NHANES) 2013–2014 database was used in this study, which may be found here: https://wwwn.cdc.gov/nchs/nhanes/search/DataPage.aspx?Component%20=%20Question-naire&CycleBeginYear%20=%202013 (accessed on 1 February 2022).

## Supplementary Materials

The following supporting information can be downloaded at: https://www.mdpi.com/article/10.3390/nu14061238/s1, Table S1: Frailty index variables, Table S2: Factor loadings of dietary patterns characteristic of olfactory dysfunction, Table S3: Multiple logistic regression analyses examining the association between dietary patterns and frailty in older adults with olfactory dysfunction, adjusted for gustatory dysfunction, Table S4: Multiple logistic regression analysis examining the association between dietary patterns and frailty in older normosmic adults, Table S5: Subgroup classified as frail by FI and robust by FP—demographics and health metrics.
