# Supplementary material for: Association of Frailty Status and Dietary Patterns in a Nationally Representative Sample of United States Adults with Olfactory Dysfunction
Source: Nutrients. 2022 Mar 15;14(6):1238. doi: 10.3390/nu14061238 (PMC8954153; doi:10.3390/nu14061238)
Supplement: Supplementary file 1 [file nutrients-14-01238-s001.zip › nutrients-1602560-SI.pdf]

nutrients

MDPI

Supplementary Materials

Association of Frailty Status and Dietary Patterns in a Nationally Representative Sample of United States Adults with Olfactory Dysfunction

Varun Vohra [1], Evelyn M. Leland [1], Rodney J. Schlosser [2], Vidyulata Kamath [3] and Nicholas R. Rowan [1,*]

Table S1. Frailty index variables.

Variable	Dataset	Code
Ever told you had a stroke	Medical Conditions	MCQ160F
Ever told you had thyroid problem	Medical Conditions	MCQ160M
Ever told you had cancer or malignancy	Medical Conditions	MCQ220
Ever told you had a heart attack	Medical Conditions	MCQ160E
Ever told you had coronary heart disease	Medical Conditions	MCQ160C
Ever told you had angina/angina pectoris	Medical Conditions	MCQ160D
Doctor ever said you had arthritis	Medical Conditions	MCQ160A
Have serious difficulty seeing	Disability	DLQ020
Have serious difficulty hearing	Disability	DLQ010
Doctor said you have diabetes	Diabetes Questionnaire	DIQ010
Ever told you had weak/failing kidneys	Kidney Conditions – Urology	KIQ022
Leak urine during physical activities	Kidney Conditions – Urology	KIQ042
Urinated before reaching the toilet	Kidney Conditions – Urology	KIQ044
Leak urine during nonphysical activities	Kidney Conditions – Urology	KIQ046
Using fork, knife, drinking from cup	Physical Functioning	PFQ061K
Dressing yourself with difficulty	Physical Functioning	PFQ061L
Getting in and out of bed with difficulty	Physical Functioning	PFQ061J
Standing up from armless chair with difficulty	Physical Functioning	PFQ061I
Managing money with difficulty	Physical Functioning	PFQ061A
Preparing meals with difficulty	Physical Functioning	PFQ061G
Standing for long periods with difficulty	Physical Functioning	PFQ061M
Stooping, crouching, kneeling with difficulty	Physical Functioning	PFQ061D
Grasping/holding small objects with difficulty	Physical Functioning	PFQ061P
Lifting or carrying with difficulty	Physical Functioning	PFQ061E
Pushing or pulling large objects with difficulty	Physical Functioning	PFQ061T
Attending social events with difficulty	Physical Functioning	PFQ061R
Experiencing confusion/memory problems	Physical Functioning	PFQ057
60 sec. pulse	Blood Pressure	BPXPLS
Systolic: Blood pres (1st rdg) mm Hg	Blood Pressure	BPXSY1
Systolic: Blood pres (2nd rdg) mm Hg	Blood Pressure	BPXSY2
Systolic: Blood pres (3rd rdg) mm Hg	Blood Pressure	BPXSY3
Serum total folate (ng/mL)	Folate Forms – Total and Individual – Serum	LBDFOT
Glycohemoglobin (%)	Glycohemoglobin	LBXGH
Red blood cell count (million cells/uL)	Complete Blood Count with 5 parts Differential - Whole Blood	LBXRBCSI
Hemoglobin (g/dL)	Complete Blood Count with 5 parts Differential - Whole Blood	LBXHGB
Red cell distribution width (%)	Complete Blood Count with 5 parts Differential – Whole Blood	LBXRDW
Lymphocyte percent (%)	Complete Blood Count with 5 parts Differential – Whole Blood	LBXLYPCT
Segmented neutrophils percent (%)	Complete Blood Count with 5 parts Differential – Whole Blood	LBXNEPCT
Number of prescription medications taken	Prescription Medications	RXDCOUNT
Taken prescription medicine, past month	Prescription Medications	RXDUSE
Ever told have osteoporosis/brittle bones	Osteoporosis	OSQ060
General health condition	Hospital Utilization and Access to Care	HUQ010
Health now compared to 1 year ago	Hospital Utilization and Access to Care	HUQ020
Overnight hospital patient in last year	Hospital Utilization and Access to Care	HUQ071

Table S2. Factor loadings of dietary patterns characteristic of olfactory dysfunction[a].

Dietary Components	Folate and Iron DP (12.2%)[b]	Calcium and Phosphorous DP (11.8%)	Magnesium and Fiber DP (11.7%)	Protein and Selenium DP (11.3%)	Carbs and Fat DP (8.5%)	β-Carotene and Vitamin-A DP (7.8%)
Protein	_	0.28	0.20	0.89	_	_
Carbs	_	_	0.17	_	-0.89	_
Total sugar	_	_	_	_	-0.66	_
Fiber	_	_	0.75	_	_	0.28
Sat. Fat	_	0.26	-0.53	_	0.31	_
MUFA	_	_	_	_	0.67	_
PUFA	_	_	0.19	_	0.56	_
Cholesterol	_	_	-0.21	0.53	_	_
Vitamin A	0.3	0.41	0.24	_	_	0.69
β-Carotene	_	_	0.31	_	_	0.94
Vitamin B1	0.67	0.29	0.134	_	_	_
Vitamin B2	0.45	0.68	_	_	_	_
Niacin	0.61	_	_	0.59	_	_
Vitamin B6	0.56	0.31	0.29	0.33	_	_
Folate	0.87	_	_	_	_	_
Vitamin C	_	0.21	0.37	_	_	0.25
Vitamin D	_	0.67	_	_	_	_
Vitamin K	_	_	0.38	0.14	_	0.59
Calcium	_	0.81	_	_	_	_
Phosphorous	_	0.76	0.36	0.36	_	_
Magnesium	_	0.39	0.79	_	0.21	0.23
Iron	0.83	_	_	_	_	_
Zinc	0.56	0.35	_	0.22	_	_
Copper	_	_	0.68	_	0.2	_
Sodium	_	_	_	0.49	_	0.24
Potassium	_	0.49	0.56	_	_	0.32
Selenium	_	_	_	0.86	_	_
Caffeine	_	_	_	_	_	_

MUFA: monounsaturated fatty acid, PUFA: polyunsaturated fatty acid. [a] Factor loadings < |0.2| were omitted. [b] Variance accounted for by each dietary pattern.

Table S3. Multiple logistic regression analyses[a] examining the association between dietary patterns and frailty in older adults with olfactory dysfunction, adjusted for gustatory dysfunction[b].

	Frailty Index			Physical Frailty		
	**AOR**	**95% CI**	**p-value**	**AOR**	**95% CI**	**p-value**
DP 1 (folate and iron)	0.93	0.84 to 1.03	0.12	1.007	0.71 to 1.42	0.95
DP 2 (calcium and phosphorus)	0.89	0.77 to 1.02	0.071	0.9	0.72 to 1.14	0.25
DP 3 (magnesium and fiber)	0.91	0.82 to 1.01	0.068	0.98	0.81 to 1.18	0.71
DP 4 (protein and selenium)	0.83	0.69 to 0.98	0.039*	0.76	0.6 to 0.97	0.036*
DP 5 (carbs and fat)	0.85	0.63 to 1.16	0.19	0.78	0.51 to 1.21	0.17
DP 6 (β-carotene and vitamin A)	0.75	0.59 to 0.97	0.036*	0.85	0.53 to 1.35	0.34

AOR: adjusted odds ratio, CI: confidence interval, DP: dietary pattern. [a] Models were adjusted for age, gender, race, BMI, income-to-poverty ratio, energy, smoking, and gustatory dysfunction (32 NaCl).[b] Methods for gustatory dysfunction classification listed in Bernstein et al. (2021) [2]. * ($p < 0.05$).

Table S4. Multiple logistic regression analysis examining the association between dietary patterns and frailty in older normosmic adults.

	Frailty Index			Physical Frailty		
	**AOR**	**95% CI**	**p-value**	**AOR**	**95% CI**	**p-value**
DP 1 (folate and iron)	1.01	0.97 to 1.04	0.73	1.0365	0.97 to 1.11	0.37
DP 2 (calcium and phosphorus)	1.00	0.97 to 1.04	0.86	1.0356	0.96 to 1.12	0.44
DP 3 (magnesium and fiber)	0.99	0.95 to 1.03	0.55	1.0072	0.94 to 1.08	0.85
DP 4 (protein and selenium)	0.97	0.92 to 1.02	0.29	0.9766	0.88 to 1.09	0.67
DP 5 (carbs and fat)	0.94	0.85 to 1.05	0.35	0.968	0.84 to 1.11	0.68
DP 6 (β-carotene and vitamin A)	0.99	0.94 to 1.04	0.70	0.98	0.93 to 1.04	0.52

AOR: adjusted odds ratio, CI: confidence interval, DP: dietary pattern. Models were adjusted for age, gender, race, BMI, income-to-poverty ratio, energy, and smoking.

Table S5. Subgroup classified as frail by FI and robust by FP—demographics and health metrics.

Age	65.9±0.62
Gender	
Male	67 (0.55)
Female	55 (0.45)
BMI	31.25±0.57
Race	
Non-Hispanic White	62 (0.51)
Mexican American	13 (0.11)
Other Hispanic	11 (0.09)
Non-Hispanic Black	27 (0.22)
Non-Hispanic Asian	7 (0.06)
Other Race	2 (0.016)
Income-to-poverty ratio	2.35±0.36
Current smoker	
No	105 (0.86)
Yes	17 (0.14)
Energy (kcal)	1862.83±28.4
Frailty index score	0.29±0.004
